# Connecting pre-existing digitalization and technology adoption speed with AI-driven business model transformation via employee competencies

**DOI:** 10.1038/s41598-026-54696-6

**Published:** 2026-05-28

**Authors:** Domitilla Magni, Sikandar Ali Qalati, Hanan Eid Badwy

**Affiliations:** 1 Department of Economics and Business Management Sciences, Catholic University of Sacre Heart, Milan, 20123 Italy; 2 Bayes Business School, City St George’s – University of London, London, UK; 3 School of Business, Liaocheng University, Shandong, Liaocheng, 252059 China; 4 Natural Resources in Environmental Systems Department Environmental Studies & Research Institute, University of Sadat City, Sadat City, Menoufia 32897 Egypt

**Keywords:** Artificial intelligence, Business model transformation, Employee competency, Digital entrepreneurship, Technology adoption, Resource-based view, Business and management, Business and management, Information systems and information technology, Science, technology and society

## Abstract

This study investigates how pre-existing digitalization and technology adoption speed shape AI-driven business model transformation, with particular attention to the mediating role of employee competencies. Grounded in the resource-based view and digital transformation theory, the study employs structural equation modeling on survey data collected from 421 employees across various industries operating in Egypt. The results reveal that pre-existing digitalization and technology adoption speed directly and indirectly influence AI-driven business model transformation. Moreover, employee competencies significantly influence AI-driven business model transformation and partially mediate the effects of pre-existing digitalization and technology adoption speed on this outcome. This work contributes to the literature on artificial intelligence and digital transformation by demonstrating that employee competencies constitute a pivotal organizational mechanism linking the technological environment to business model innovation. Further, the study offers empirical insights into an emerging economy where the interplay between human capital and technological advancement has not been adequately studied.

## Introduction

Artificial intelligence (*AI*) is not only reshaping technological capabilities but also redefining how firms adapt, evolve, and learn in the era of dynamic change^69^. Yet, beyond its technical potential, AI prompts organizations to rethink internal processes and learning mechanisms, especially when early setbacks or implementation challenges arise during the transformation journey^61^. Furthermore, the rapid evolution of AI technologies has led to substantial transformations across numerous sectors globally. The adoption of AI-driven systems has become a key factor in business model redesign, offering innovative solutions to meet customer needs^64,65^. AI-driven business model transformation (*BMT*) is therefore the integration of AI technologies into the fundamental aspects of an organization’s activities, strategic framework, and value proposition. The technologies enable firms to automate tasks, augment functions, and improve decision-making^30,31^. The integration of AI into business processes requires a nuanced understanding of how digital transformation reshapes organizational structures. Digital technologies do not merely replace existing systems but fundamentally reconfigure decision-making processes and managerial dynamics^26^.

Organizations aiming to achieve digital transformation require an understanding of the organizational environment that enables successful AI integration, and employee competence is now considered crucial^4^. The interplay between digitalization and business models necessitates a strategic approach in which organizations leverage digitalization not only for operational efficiency but also to reimagine value chains and market positioning^1^. In addition, the employee competence level (*ECL*) is identified as an essential driver of the successful deployment of advanced technologies. Competency is not only technical, such as the ability to use software or perform data analysis, but also behavioral, involving flexibility and problem-solving skills^6,8^. Furthermore, research by Farayola et al.,^22^ found that employees’ ability to successfully interact with and leverage these advanced technologies is central to a successful transition to an AI-driven business model. In sectors with pre-existing digitalization (*PED*), employees are more inclined to have the skills required to adopt advanced technologies, including AI^65^.

PED determines the extent to which firms have already embraced digital technologies and systems before AI technologies were developed^10,34^. A workforce exposed to digital technologies is more likely to understand the broader digital ecosystem and be more receptive to the changes ushered in by AI systems^3^. The pace of technology adoption can also spur employees to rapidly enhance their skills and learn, resulting in an overall improvement in their competencies^36^. However, an accelerated pace of technology adoption is argued to generate significant challenges, including gaps in employee training, resistance to change, and a continuous need for support and upskilling factors that underscore the critical importance of a well-calibrated and strategic approach to technology implementation^4,42^.

The introduction of AI creates opportunities but also brings uncertainties and misalignments^56^. It is necessary to create internal systems within an organization to identify and interpret emerging signals of failure during the transformation process^58^. Adopting AI technology in business processes entails significant risks, as technological complexities and skills shortages can lead to failure rather than successful transformation^11^. Identifying how businesses can overcome setbacks using their internal capabilities is crucial to avoiding the negative effects of AI technologies^41^. Adopting AI technology in business processes also increases the risk of failure due to a lack of employee skills^57^.

Although there is past work on the effects of AI on business model transition, little is known about the competencies of workers who facilitate this process. Moreover, most works in the field focus on Western contexts and neglect other regions, such as Egypt, where digital transformation is challenging due to the region’s nature. As Egypt has a young population, and technological advancements and innovations are developing in the country, it will be interesting to see how the digital transformation proceeds in Egypt. Recently, the Egyptian authorities have launched various programs to encourage the adoption of digitalization and AI across sectors. However, many enterprises in Egypt face digital skills gaps and lack adequate infrastructure. In this regard, the current study seeks to fill a significant gap in the literature by analyzing the effect of employee competencies on the interaction between PED, technology adoption speed (TAS), and AI-driven BMT. Whereas existing literature has sought to debate digitalization and innovation in business models^43,50,67^, this research focuses on the AI-driven work as the role of ECL as a mediator between PED, TAS, and AI-driven BMT.

By applying the lens of the resource-based view (RBV)^5^, the research offers new insights into the organizational dynamics required to successfully implement AI in business models. In the context of digital transformation, the RBV remains relevant, since the adoption of emerging technologies, such as AI, requires not only technological investments but also qualified human resources and specific skills^53^. Employee competencies emerge as a strategic resource that mediates the impact of digitization and technology adoption on business model transformation^28^. The present work adds to the ongoing academic discussion by examining the intricate link among PED, TAS, and ECL in the context of business model transformation, specifically across the industries present in Egypt (e.g., manufacturing). The findings of this study make a significant theoretical contribution by integrating the RBV with the literature on digital transformation. Furthermore, they provide practical guidance for managers, highlighting the significance of investing in employee training and accelerating technology adoption to realize innovative and sustainable business models.

## Literature review and hypotheses development

### Underpinning theory

The RBV, introduced by Barney^5^, is one of the most influential theoretical frameworks in strategic management. In the RBV, the firm’s competitive advantage derives from internal resources and competencies. These resources, whether tangible or intangible (e.g., employee skills), form the foundation for building dynamic capabilities^49^.

Considering the digital transformation, RBV gains renewed relevance, as the integration of emerging technologies such as AI demands more than infrastructure and tools; it requires skilled human capital capable of leveraging these technologies effectively^21^. ECL, therefore, emerges as a pivotal strategic resource that can mediate the effects of PED and TAS on AI-driven BMT. This perspective is particularly pertinent when organizations face the high uncertainty and complexity inherent in AI-related innovation, where failure is not uncommon^47^. The ability to learn from implementation challenges and early setbacks is increasingly recognized as a strategic differentiator^9,15^. In this light, employee competencies contribute not only to facilitating adoption but also to fostering organizational learning from AI-driven innovation processes, turning potential points of failure into opportunities for business model renewal.

Recent studies have highlighted how the adoption of emerging technologies, such as AI, is redefining business models in various industries^17^. However, most of this research has focused on Western contexts, neglecting the unique dynamics of emerging countries, where factors such as digital infrastructure availability, ECL, and the institutional environment can vary significantly. Moreover, although the RBV literature has extensively explored the role of organizational resources^5^, few studies have applied this perspective to AI-driven digital transformation. This gap provides an opportunity to contribute to the existing literature by integrating RBV with studies on digitization and technological innovation.

In this research, employee competencies were identified as a key resource that significantly influences the adoption and effective use of AI technologies. This approach aligns with the idea that employee competencies and knowledge, which can be developed through systematic training and experiential learning, are critical to the success of AI-driven BMT^19^. Digital skills enable employees to interact productively with new technologies, facilitating the implementation of innovative processes and value creation. In addition, PED can be seen as an independent asset that supports organizations in using AI technologies more effectively^63^. Robust and well-integrated digital infrastructures provide a solid foundation for implementing advanced solutions, reducing adoption time, and improving operational efficiency. However, without appropriate skills, even the most advanced infrastructure risks remain underutilized or fail to reach their full potential.

RBV provides a theoretical framework for explaining how employee competencies and digital infrastructure complement each other to facilitate AI-driven adoption and transformation. Specifically, the theory suggests that organizations that invest in both technology resources and human skills development are better positioned to meet the challenges of digital transformation and to take advantage of the opportunities presented by emerging technologies. This integrated approach enables overcoming the limitations of a purely technological view by recognizing the fundamental role of human resources as a driver of innovation and organizational change.

### PED and ECL’s relationship

Employee competencies and skills can be divided into several categories, including technical skills, e.g., programming, data analysis, and computer systems management, essential for interacting directly with advanced technologies; digital skills, e.g., using digital tools, managing online platforms, understanding data flows, enabling employees to navigate effectively in digitized environments; adaptive skills, e.g., flexibility, continuous learning, resilience to change, essential for coping with rapid technological and organizational change. In line with the RBV^5^, employee competencies and skills represent an intangible resource that is difficult to replicate and can be enhanced through investments in PED.

Recent studies have shown that organizations with highly skilled employees are more likely to successfully implement advanced technologies and innovate their business models^7^. This is because skilled employees are not only able to use new technologies effectively but can also actively contribute to their optimization and integration into business processes. In addition^36^, reports that continuous exposure to digital tools increases employees’ familiarity with technology, facilitating the acquisition of more advanced skills. PED provides a continuous learning platform where employees can experiment with their digital skills in a real-world operational context. As employees become familiar with digital environments, they develop higher digital literacy and greater ability to use the latest technologies, thereby improving their overall competence^62^. Experience in a digitized work environment improves their understanding of technological innovations, preparing them to more effectively meet the challenges of adopting advanced tools, such as artificial intelligence^65^. Thus, PED plays a key role in enhancing employee skills. Established digital infrastructure and an already technology-driven work environment offer employees the opportunity to develop basic digital skills, which serve as a springboard for acquiring more advanced skills. This incremental learning process enables employees to adapt more quickly to new technologies and actively contribute to the organization’s digital transformation. Thus, hypothesize that.

#### H1

*PED has a positive impact on ECL because it provides a continuous learning environment that facilitates the acquisition and refinement of digital and technical skills.*

### ECL and AI-driven BMT’s relationship

Employees with a higher ECL demonstrate an exceptional ability to understand, integrate, and implement AI technologies, facilitating a smooth transition and meaningful transformation within the organization^62^. According to Davenport and Ronanki^17^, employees’ digital skills are a critical factor in implementing AI solutions, as they enable the full potential of these technologies to be realized. These employees are not only able to use AI technologies effectively but can also actively contribute to their optimization, adapting them to the organization’s specific needs and maximizing their potential. In line with the RBV, employee skills represent a resource that can be harnessed to create a sustainable competitive advantage through technological innovation^52^. Advanced employee skills translate into tangible improvements in the productivity, efficiency, and quality of business processes. For example, highly skilled employees can use predictive analysis tools to optimize the supply chain or implement machine learning algorithms to personalize the customer experience. In addition, their problem-solving and critical thinking skills improve decision-making, enabling organizations to respond more quickly and more effectively to market challenges^4^.

A particularly relevant aspect is the adaptability of competent employees. In an environment characterized by rapid technological change, flexibility and the willingness to learn new skills are key to maintaining a company’s competitiveness. Employees with a high level of competence not only adapt more easily to new technologies but are also able to anticipate future trends and propose innovative solutions. This makes them a key factor in the success of AI-based operations and the transformation of business models^65^. Furthermore, employee competencies play a crucial role in overcoming the resistance to change often associated with the adoption of advanced technologies. Competent employees can act as ‘digital ambassadors’, promoting an innovation-oriented corporate culture and facilitating the adoption of new practices and tools. This contributes to creating an organizational environment that fosters experimentation and continuous learning, which are essential for the success of digital transformation^19^. Thus, hypothesize that.

#### H2

*ECL has a positive impact on AI-driven BMT*,* as highly skilled employees facilitate the integration and optimization of AI technologies*,* improve decision-making processes*,* and promote an innovation-oriented organizational culture.*

### PED and AI-driven BMT’s relationship

Organizations that have already embarked on a PED path are well-positioned to adopt more advanced technologies, such as AI. The presence of established digital systems, optimized processes, and robust technological infrastructure provides a solid foundation for building new digital transformation initiatives^33^. This pre-existing infrastructure not only reduces the cost and time required to implement AI technologies but also facilitates the integration of these solutions into existing workflows, minimizing operational disruptions and maximizing efficiency. A crucial aspect of PED is its impact on organizational culture and employee mentality. Hence, AI-driven transformations are deeply embedded in socio-cultural and institutional contexts, requiring organizations to not only adopt new technologies but also redefine their strategic narratives to align with evolving digital ecosystems^60^. Organizations that are already digitized tend to develop a corporate culture that is more open to innovation and change, in which employees are accustomed to using digital tools and experimenting with new technologies. This creates a favorable environment for the adoption of AI solutions, as employees are more likely to see these technologies as a natural extension of existing processes rather than a radical revolution^2^. In addition, PED provides employees with greater familiarity with digital platforms, increasing their confidence and willingness to adopt AI solutions. This familiarity reduces resistance to change and facilitates the acquisition of new skills, accelerating the adoption and integration process of AI technologies. As a result, organizations with a strong digital foundation are better positioned to exploit the full potential of AI, transforming their business models more quickly and effectively^65^.

A further advantage of PED is its ability to generate structured, accessible data, a key resource for implementing AI solutions. Established digital infrastructures allow large volumes of data to be collected, stored, and analyzed, providing the ‘fuel’ needed to power machine learning algorithms and other advanced technologies. This creates a virtuous circle in which pre-existing digitization not only facilitates the adoption of AI but also amplifies its impact, enabling organizations to gain insights and make more informed decisions^19^. Thus, hypothesize that.

#### H3

*PED has a positive impact on AI-driven BMT by providing an infrastructural and cultural foundation that facilitates the adoption*,* integration*,* and optimization of AI technologies*,* thereby accelerating digital transformation.*

### Relationship between TAS and ECL

The rapid adoption of new-generation technologies requires employees to adapt to an accelerated pace of technological change, necessitating the development of new skills and, in particular, high adaptability^55^. This accelerated adoption process not only pushes employees to quickly familiarise themselves with advanced tools and systems but also exposes them to a dynamic learning environment, significantly shortening the learning curve^18^. As a result, employees acquire greater technological competence and practical familiarity with digital innovations, resulting in a better ability to use modern technologies.

A key aspect of this report is TAS’s role in creating opportunities for continuous learning. Rapid technological adoption exposes employees to a continuous stream of new tools and processes, fostering the development of advanced competencies such as digital literacy, analytical thinking, and technical proficiency^2^. This process of continuous exposure not only enhances existing skills but also encourages the acquisition of new ones, making employees more versatile and prepared to face the challenges of an ever-changing digital environment. Moreover, accelerated technology adoption creates a virtuous circle in which employees, once they have acquired the necessary skills, become more inclined to experiment and innovate, thereby further contributing to the organization’s digital transformation^65^. Thus, hypothesize that.

#### H4

*The TAS has a positive impact on ECL*,* as an accelerated pace of adoption exposes employees to a dynamic learning environment*,* stimulating the development of digital*,* analytical*,* and technical skills.*

### Relationship between TAS and AI-driven BMT

The rapid adoption of AI technologies facilitates the accelerated integration of advanced tools into organizational processes, enabling companies to innovate and transform their business models more efficiently^39^. This rapid adoption process not only reduces implementation time but also enables organizations to immediately reap the benefits of AI technologies, such as improved operational processes, resource optimization, and new market opportunities. A crucial aspect of TAS is its ability to generate sustainable competitive advantage. Organizations that rapidly adopt AI solutions can anticipate market trends, respond more quickly to customer needs, and differentiate themselves from competitors^13^. For example, the timely implementation of machine learning algorithms can improve supply chain efficiency, while the use of predictive analytics tools can optimize marketing and sales strategies. Moreover, the rapid adoption of AI technologies enables organizations to optimize operational processes more effectively, reducing costs and improving the quality of the products or services they offer. This translates into a better customer experience and a greater ability to develop innovative value propositions^30^. For example, companies that rapidly adopt AI-based chatbots can improve customer service, while using advanced recommender systems can personalize business offerings, increasing customer satisfaction and loyalty. However, the impact of TAS on AI-driven BMT is not only operational but also strategic. Rapid adoption allows organizations to experiment and iterate faster, identify new business opportunities, and adapt to market changes in real time. This dynamic approach is particularly relevant in competitive environments, where the ability to innovate quickly can be a critical success factor^65^. Thus, hypothesize that.

#### H5

*TAS has a positive impact on AI-driven business BMT*,* as rapid adoption enables organizations to optimize operational processes*,* improve customer experience*,* and develop innovative value propositions*,* accelerating digital transformation.*

### The mediating role of ECL

Organizations with a strong portfolio of digital systems and technologies tend to foster a corporate culture in which employees develop greater technological familiarity and competence. This digitally advanced environment not only enhances employees’ digital capabilities but also increases their propensity to innovate and experiment with new technologies, such as AI^32^. In other words, PED creates an environment in which employees can hone their skills, becoming more aware, open, and capable of using advanced technologies. This enhanced ECL plays a crucial role in facilitating the AI-driven BMT. Highly competent employees are not only able to understand and implement AI solutions but can also actively contribute to their optimization by adapting them to the organization’s specific needs. In this way, employee competencies act as a bridge between pre-existing digitization and business model transformation, accelerating adoption and maximizing impact^6^.

A particularly relevant aspect is the role of employees’ competencies in overcoming barriers to adopting AI technologies. Competent employees can act as change agents, promoting an innovation-oriented corporate culture and reducing the resistance to change often associated with the introduction of new technologies. This contributes to an organizational environment that fosters experimentation and continuous learning, which are essential elements for successful digital transformation^65^. Furthermore, employee skills enable organizations to fully exploit the potential of PED. Established digital infrastructures, when combined with a highly competent workforce, can generate a synergy effect that amplifies the impact of AI technologies. For instance, competent employees can use structured, accessible data to develop more sophisticated machine learning algorithms, thereby improving operational efficiency and creating new business opportunities^19^. Thus, hypothesize that.

#### H6

*ECL mediates the relationship between PED and AI-driven BMT*,* as a highly competent workforce facilitates the integration and optimization of AI technologies*,* accelerating digital transformation and maximizing its impact.*

**Fig. 1:**
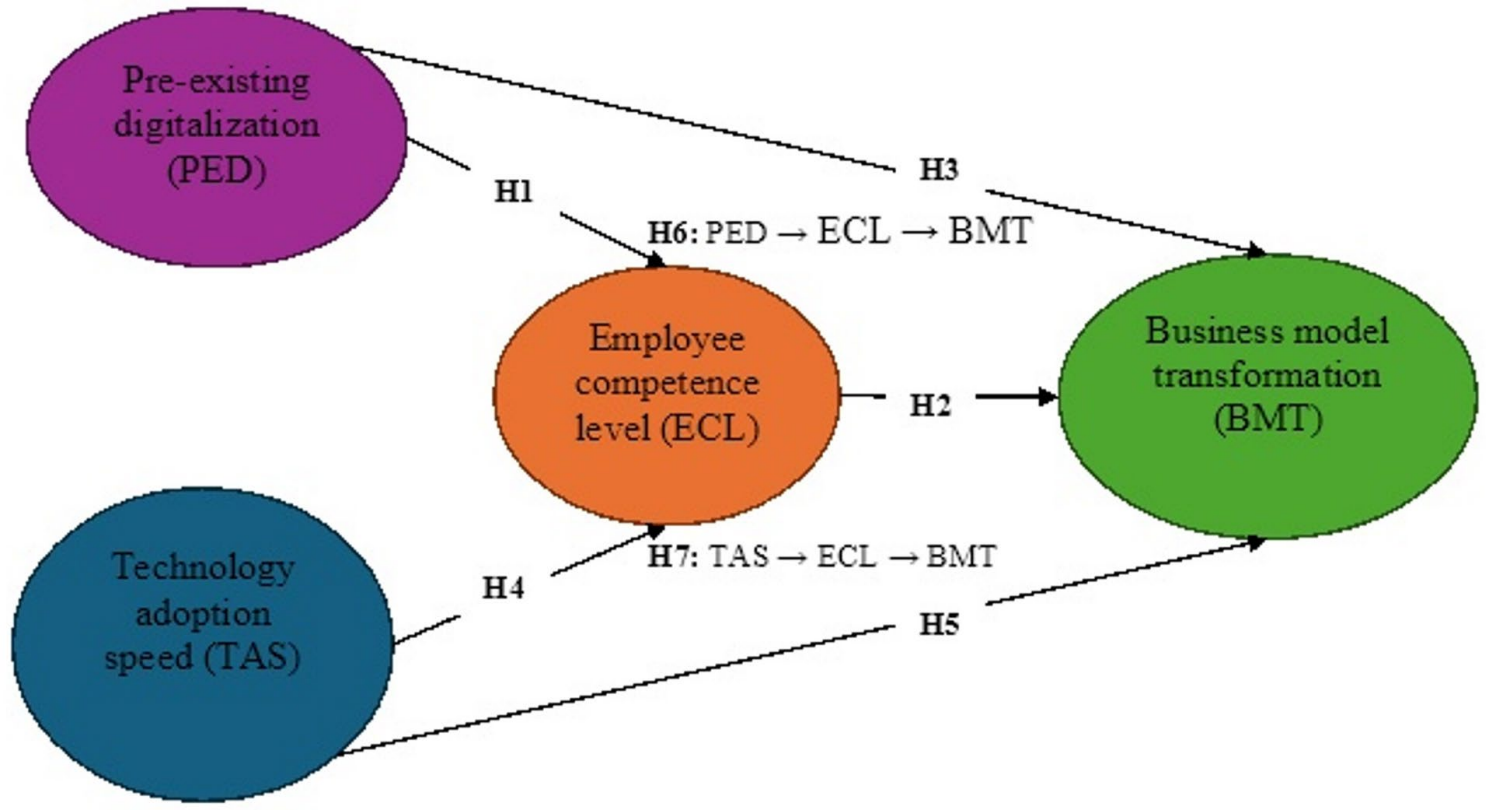
Research Model. Source: Author’s development.

The adoption of emerging technologies requires employees to learn and adapt at an accelerated pace, developing the skills needed to use advanced tools and systems effectively. In this context, ECL plays a crucial role in mediating the relationship between the TAS and AI-driven BMT. Highly competent employees are not only able to quickly understand and implement AI technologies but also to actively contribute to their optimization, thereby accelerating their integration into organizational processes^42^. A key aspect of this mediation is employees’ ability to translate technology adoption into tangible results. When employees possess advanced skills, they can exploit the full potential of AI technologies, improving operational processes, optimizing resources, and creating new business opportunities. Conversely, in the absence of appropriate skills, even rapid technology adoption may not yield the desired effects, as employees may be unable to effectively use the tools available to them^45^. Furthermore, competent employees act as agents of change, fostering an innovation-oriented corporate culture and reducing the resistance to change often associated with the introduction of new technologies. This contributes to an organizational environment that fosters experimentation and continuous learning, both essential to the success of digital transformation^65^. Thus, hypothesize that (Fig. 1).

#### H7

*ECL mediates the relationship between TAS and AI-driven BMT*,* as highly competent employees facilitate the integration and optimization of AI technologies*,* accelerating digital transformation and maximizing its impact.*

## Methodology

### Sampling and data collection

The target population of this study consists of employees in key industrial sectors operating in Egypt. The sectors selected for analysis include manufacturing, services, technology, finance, retail, and healthcare, chosen for their significant contribution to the country’s economic framework and their impact on the labor market. These sectors are key pillars of economic growth^51^, employment generation, and overall development in Egypt. In addition, they exhibit varying levels of digitization, rates of technological integration, AI adoption, business model innovation, and workforce skills, making them ideal for a comprehensive analysis of the proposed research hypotheses.

Egypt provides an ideal context for this study due to its strategic role as an emerging economy in North Africa and the Middle East. With a young, fast-growing population, an expanding technology sector, and government initiatives to promote digitization, Egypt offers a natural laboratory for exploring the dynamics of AI-driven digital transformation. Moreover, the country’s diverse industries, ranging from traditional manufacturing to high-tech services, enable analysis of how employee skills and the adoption of advanced technologies influence business models across contexts. The Egyptian government has recently launched several initiatives, such as Vision 2030, which aims to transform the country into a regional digital hub, stimulating the adoption of emerging technologies and promoting business innovation. Despite these efforts, many organizations still face significant challenges, such as inadequate digital infrastructure and a lack of specialized skills, making Egypt a relevant case study for exploring the barriers and opportunities of digital transformation.

To ensure a broad and representative geographical coverage, the study adopted a non-probability sampling approach for convenience. This method was chosen for its convenience and the ability to reach many respondents across different regions of Egypt. Data were collected via online questionnaires and distributed through LinkedIn profiles linked to the various target sectors. The questionnaires were designed to include questions on pre-existing digitization, the speed of technology adoption, employee skills, and business model transformation. Subsequently, the data were analyzed using partial least squares structural equation modeling (PLS-SEM), which is particularly suitable for exploratory studies and complex models with many variables^12^. The research design is based on cross-sectional data, which offers significant opportunities for conceptual knowledge development and theory advancement, especially in emerging contexts such as Egypt^46^. 500 questionnaires were distributed, of which 421 responses were considered valid and usable, with a response rate of 84.2%. The final sample of 421 observations is adequate to meet the minimum requirement suggested by Pesämaa et al.,^46^ of at least 400 responses (40 items x 10 responses per item) to ensure the robustness of the statistical analysis.

### Survey design

The instruments used in this research were rated using five-point Likert scales, ranging from 1 (‘Strongly disagree’) to 5 (‘Strongly agree’). Five-point Likert scales were chosen for their balance between simplicity and ability to capture the nuances of responses. This format is widely recognized for reliably measuring attitudes and perceptions and is particularly suitable for studies exploring complex phenomena such as digital transformation^20^.

The PED dimension was measured using 10 items adapted from Westerman et al.,^68^, who developed a framework to assess corporate digitization, and Kraus et al.,^33^, who analyzed the impact of pre-existing digitization on the adoption of emerging technologies. Additionally, the TAS was measured through ten items, formulated based on research by Liu and Jiang^37^ and Day and Schoemaker^18^, which explored how the speed of technology adoption affects innovation and business competitiveness. Moreover, the ECL’s ten items, adapted from the studies by Di Vaio et al.,^19^ and Trenerry et al.,^65^, which analyzed the role of digital and technical competencies in digital transformation, were used to measure the ECL. Lastly, the AI-driven BMT was measured through ten items, adapted from the work of Davenport and Ronanki^17^, Ji et al.,^30^, and Magni et al.,^40^, who explored how artificial intelligence is redefining business models in various industries. All items adapted from previous studies are listed in Appendix A.

To ensure the validity and reliability of the instruments, a pretest was conducted on a pilot sample of 30 participants, followed by an analysis of internal consistency (Cronbach’s alpha) and convergent and discriminant validity. The pretest confirmed the clarity and relevance of the items, while the internal consistency analysis showed Cronbach’s alpha values above 0.80 for all dimensions, indicating a high reliability of the instruments.

## Results

### Sample profile

#### Industry profile

The organizations included in the study present a diverse profile across several sectors, with significant representation in technology, services, and finance. These sectors, known for their high rates of innovation and adoption of advanced technologies, provide an ideal context for exploring the impact of AI on business model transformation. The technology sector is known for its pioneering adoption of AI solutions, while financial services are leveraging AI to optimize decision-making and improve the customer experience. Likewise, service-driven organizations are increasingly incorporating AI into managerial and operational processes to improve efficiency, responsiveness, and value creation.

#### Firm size and firm capacity

Most of the companies in the sample belong to the large enterprise group, which comprises companies with more than 250 employees and annual revenue between EUR 50–100 million. Being a large enterprise means that the company has the potential to deploy sophisticated technologies and pursue a wide range of digital transformations. Indeed, as a large enterprise with strong financial capabilities and the ability to attract talented specialists, the organization is likely to play a proactive role in implementing AI technologies. This aspect is especially relevant to the current study, as AI-driven BMT often requires significant investment.

#### Geographic profile and Egyptian context

Geographically, firms operating in Africa and Asia make up the bulk of the sample, accounting for 63.2% of all cases. Nevertheless, it needs to be highlighted that this research is carried out in an Egyptian setting based on the location of companies and respondents and not based on the headquarters of companies. There are some multinational companies with headquarters in Africa, Asia, North America, Europe, and even South America, but with operations in Egypt for this particular study. Therefore, Table 1 presents the continents with reference to the parent company headquarters.

The above difference is important to this study because it examines firms in Egypt, either local firms or branches of firms headquartered elsewhere. The reason Egypt is used in this case is that it serves as a regional technology center, making it an appropriate context for discussing digital transformation in a rapidly growing economy. At the same time, since some of the sampled companies are multinational, care needs to be taken when generalizing.

#### AI-related roles and investment profile

Concerning investments in AI, the companies in the sample show significant commitment, with AI Specialists and R&D/Innovation Specialists occupying a central role. Most companies have been undertaking AI-based research and development (R&D) activities for more than five years, allocating more than 50% of their R&D investments to AI-related initiatives. This level of investment reflects the growing awareness of AI’s potential to drive innovation and business competitiveness. According to Iansiti and Lakhani^29^, companies that invest in AI tend to gain a significant competitive advantage by improving operational efficiency and creating new business opportunities.

The inclusion of participants holding roles related to AI implementation, innovation management, and R&D makes the sample more appropriate for studying how technological capabilities and employee competencies drive changes in business models through AI.

#### Overall suitability of the sample

These characteristics confirm AI’s pioneering role in business development and innovation, not only within specific sectors but also across geographies. Simultaneously, it should be noted that since some of the companies involved in the study operate internationally, one must be careful when claiming that all firms in Egypt can be regarded in the same way. Nevertheless, as some of them are known for their clear preference for AI and their involvement in research and development, it becomes evident that AI is important to the global digitalization process.

The sample provides a suitable empirical context for studying the impact of AI on the transformation of business models among firms based in Egypt, while taking into account the constraints associated with diversity in firm origins and the sampling methodology used in the research.

**Table 1:** Industry profile.

	Item	Frequency	%
The company’s industry sector	Manufacturing	34	8.1
	Services	96	22.8
	Technology	93	22.1
	Finance	115	27.3
	Retail	44	10.5
	Healthcare	25	5.9
	Other	14	3.3
Number of employees	Small (< 50 employees)	52	12.4
	Medium (50–249 employees)	169	40.1
	Large (+ 250 employees)	200	47.5
The company’s annual revenue	< €10 million	19	4.5
	€10–50 million	72	17.1
	€50–100 million	178	42.3
	> €100 million	152	36.1
Company’s headquarter	Europe	29	6.9
	North America	81	19.2
	South America	22	5.2
	Asia	111	26.4
	Africa	155	36.8
	Oceania	23	5.5
Job role	Executive/CEO/Founder	42	10.0
	Manager	110	26.1
	R&D/Innovation Specialist	141	33.5
	IT/AI Specialist	121	28.7
	Other	7	1.7
Years of investment in AI-related R&D	Less than 1 year	19	4.5
	1–3 years	21	5.0
	3–5 years	88	20.9
	More than 5 years	293	69.6
% of company’s total R&D budget allocated to AI	< 10%	19	4.5
	10–25%	23	5.5
	25–50%	167	39.7
	> 50%	212	50.4

Source: Author’s development.

### Measurement model evaluation

In the model evaluation phase, constructs were systematically analyzed using three key indicators: composite reliability (CR), Cronbach’s alpha (CA), and average variance extracted (AVE), following the guidelines proposed by Sarstedt et al.,^54^. These indicators are widely recognized in the methodological literature to assess the reliability and convergent validity of constructs in complex structural models. According to Hair et al.,^27^, the combined use of CR, AVE, and VIF is considered a best practice to assess the reliability and validity of measurement models in PLS-SEM-based studies.

**Fig. 2:**
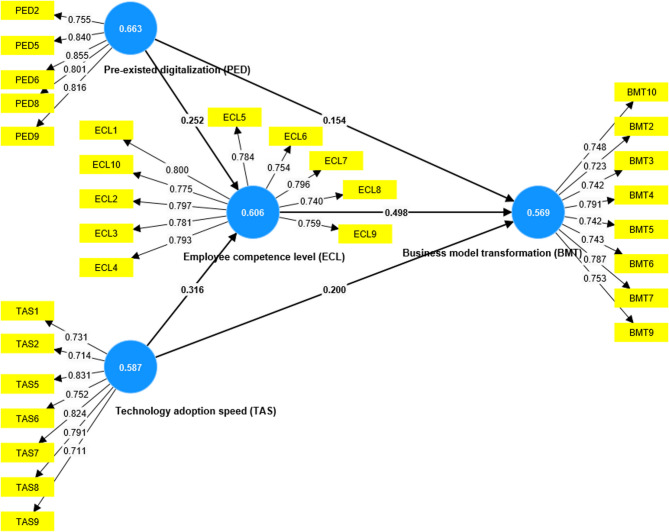
Measurement model evaluations.

As shown in Table 2, excepts items represented by asterisk value (PED1, PED3, PED4, PED7, PED10, TAS3, TAS4, TAS10, BMT1, and BMT8), all items achieved factorial loadings above the minimum threshold of ≥ 0.701, as recommended by Hair et al.,^27^. Figure 2 presents the results of the measurement model, including factor loadings and AVE values. This result confirms that each item contributes significantly to measuring the corresponding construct. Furthermore, both CA and CR exceeded the recommended minimum values of 0.701, demonstrating high internal consistency and construct reliability^27^. Similarly, the AVE exceeded the minimum threshold of 0.50 for all constructs, confirming the convergent validity of the model^27^(see Fig. 2).

To assess multicollinearity, the variance inflation factor (VIF) was calculated for each construct. The VIF values remained consistently below the maximum acceptable threshold of 3.3, as suggested by Sarstedt et al.,^54^, indicating no excessive correlation among constructs and that the model is statistically robust.

**Table 2:** The reliability and validity analysis.

Construct	Item	Loadings	CA	CR	AVE	VIF
Pre-existing digitalization (PED)	PED1	0.597^*^	0.872	0.876	0.663	2.314
	PED10	0.645^*^				
	PED2	0.755				
	PED3	0.699^*^				
	PED4	0.586^*^				
	PED5	0.840				
	PED6	0.855				
	PED7	0.567^*^				
	PED8	0.801				
	PED9	0.816				
Technology adoption speed (TAS)	TAS1	0.731	0.882	0.885	0.587	2.225
	TAS10	0.687^*^				
	TAS2	0.714				
	TAS3	0.549^*^				
	TAS4	0.669^*^				
	TAS5	0.831				
	TAS6	0.752				
	TAS7	0.824				
	TAS8	0.791				
	TAS9	0.711				
Employee competence level (ECL)	ECL1	0.800	0.928	0.932	0.606	1.392
	ECL10	0.775				
	ECL2	0.797				
	ECL3	0.781				
	ECL4	0.793				
	ECL5	0.784				
	ECL6	0.754				
	ECL7	0.796				
	ECL8	0.740				
	ECL9	0.759				
Business model transformation (BMT)	BMT1	0.681^*^	0.893	0.896	0.569	
	BMT10	0.748				
	BMT2	0.723				
	BMT3	0.723				
	BMT4	0.791				
	BMT5	0.742				
	BMT6	0.743				
	BMT7	0.787				
	BMT8	0.636^*^				
	BMT9	0.753				

Note: * represents value removed.

*Source*: Author’s development.

Table 3 shows that all constructs’ heterotrait-monotrait (HTMT) ratios fall below the 0.85 and 0.90 suggested cutoff^27^. Additionally, the results also meet the^23^ criterion as the √AVE for each variable (bold and diagonal values) exceeds the corresponding inter-factor correlation.

**Table 3:** Discriminant validity analysis.

Variables	HTMT analysis				Fornell-Larcker analysis			
	BMT	ECL	PED	TAS	BMT	ECL	PED	TAS
BMT					*0.754*			
ECL	0.732				0.673	*0.778*		
PED	0.587	0.521			0.544	0.486	*0.814*	
TAS	0.595	0.530	0.841		0.564	0.503	0.742	*0.766*

Note: Italic values on the diagonal represent the square root of the AVE.

*Source*: Author’s development.

### Structural model evaluation

The evaluation structural model involved computing several criteria. (1) We used *R*^*2*^ (coefficient of determination). It reflects the explanatory power of the variable on the dependent variable. Table 4 reveals that PED and TAS jointly explain 28.1% of the variance in ECL, and, when combined with ECL, these three factors explain 53.2% of the variance in BMT, exceeding the 10% cutoff^27^.

**Fig. 3:**
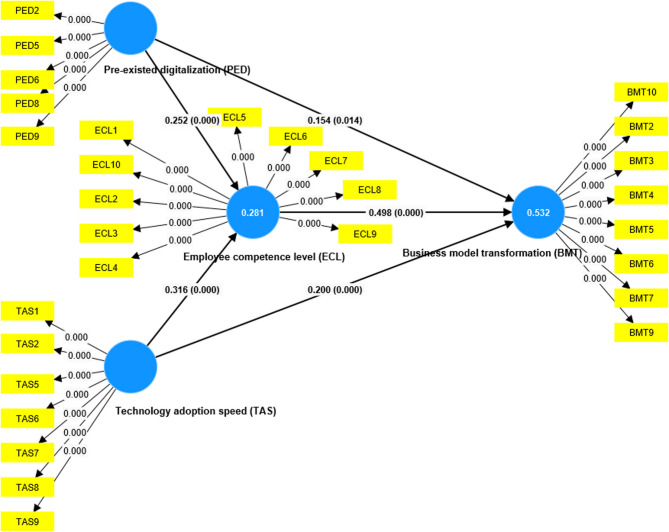
Structural model evaluation.

(2) We considered the effect size (*f*^*2*^), which measures the strength of the relationships. Table 4 indicates the large effect of ECL on BMT (0.381 > 0.35), while the other effects among the relationships were small, such as PED on ECL (0.040), on BMT (0.022), TAS on ECL (0.062), and on BMT (0.036), which are above 0.02^14^.

(3) We also utilized predictive relevance (*Q*^*2*^), which demonstrates the model’s accuracy. The analysis revealed a predictive relevance value of 0.287 for BMT, which exceeds the cutoff of zero, reflects higher predictive relevance^27^, indicating adequate relevance of the model.

(4) Table 4; Fig. 2 indicate that PED positively influences ECL (*β* = 0.252, *p* = 0.000) and BMT (*β* = 0.279, *p* = 0.000); thus, *H1* and *H3* are supported. Additionally, ECL positively affects BMT (*β* = 0.498, *p* = 0.000), indicating that higher ECL is associated with higher BMT. Therefore, *H2* is supported. Further, TAS also positively affects ECL (*β* = 0.316, *p* = 0.000) and BMT (*β* = 0.357, *p* = 0.000); thus, *H4* and *H5* are supported. Additionally, the results also supported a significant mediating impact of ECL between PED and BMT (*β* = 0.125, *p* = 0.000) and between TAS and BMT (*β* = 0.157, *p* = 0.000); thus, *H6* and *H7* are supported (Fig. 3).

**Table 4:** Assessment of the hypothesis.

Hypothesis	Relationships	βeta	t-value	*p*-value	Decision	f^2^
*Direct effect*						
H1	PED → ECL	0.252^***^	4.196	0.000	Supported	0.040
H2	ECL → BMT	0.498^***^	9.734	0.000	Supported	0.381
H3	PED → BMT	0.279^***^	4.010	0.000	Supported	0.022
H4	TAS → ECL	0.316^***^	5.282	0.000	Supported	0.062
H5	TAS → BMT	0.357^***^	5.675	0.000	Supported	0.036
*Indirect effect*						
H6	PED → ECL → BMT	0.125^***^	3.900	0.000	Supported	
H7	TAS → ECL → BMT	0.157^***^	4.634	0.000	Supported	

*Notes*: ^***^p < 0.001. PED = pre-existing digitalization; ECL=employee competence level; BMT=business model transformation; TAS=technology adoption speed. *Q*^*2*^= ECL (0.162) and BMT (0.287).

### Mediation effects of ECL

The results yield that both indirect effects are statistically significant because the bootstrapped confidence intervals do not include zero (see Table 5). Moreover, the VAF values indicate that the mediations in both cases are partial, given that they are 0.448 (44.8%) and 0.439 (43.9%), both within the 20–80% range^27^. This indicates that the mediator, ECL, accounts for part of the relationship between PED/TAS and BMT.

**Table 5:** Mediation effects of ECL on BMT.

	Path a PED→ECL	Path b ECL→BMT	Indirect effect	VAF test $\:\frac{\boldsymbol{I}\boldsymbol{n}\boldsymbol{d}\boldsymbol{i}\boldsymbol{r}\boldsymbol{e}\boldsymbol{c}\boldsymbol{t}\:\boldsymbol{e}\boldsymbol{f}\boldsymbol{f}\boldsymbol{e}\boldsymbol{c}\boldsymbol{t}}{\boldsymbol{T}\boldsymbol{o}\boldsymbol{t}\boldsymbol{a}\boldsymbol{l}\:\boldsymbol{e}\boldsymbol{f}\boldsymbol{f}\boldsymbol{e}\boldsymbol{c}\boldsymbol{t}}$	Confidence Interval		Decision
					95% LL	95% UL	
PED→ECL→BMT	0.252 (0.000)	0.498 (0.000)	0.125 (0.000)	$\:\frac{0.125}{0.279}=0.448$	0.065	0.190	Partial mediation
	*Path a* *TAS→ECL*	*Path b* *ECL→BMT*	*Indirect effect*		*Confidence Interval*		
TAS→ECL→BMT	0.316 (0.000)	0.498 (0.000)	0.157 (0.000)	$\:\frac{0.157}{0.357}=0.439$	0.092	0.226	Partial mediation

Note: PED = pre-existing digitalization; ECL=employee competence level; BMT=business model transformation; TAS=technology adoption speed.

Source: Author’s development.

## Discussion and implications

The findings underscore the urgent need for further research into integrating AI-driven business models within organizations. Specifically, the results confirm that PED significantly enhances ECL (*H1*), which aligns with the findings of Szabó et al.,^62^ and Trenerry et al.,^65^. These studies suggest that a strong foundation in digitalization equips employees with the necessary skills and confidence to engage with emerging technologies, ultimately improving their overall competency.

Moreover, the findings indicate that ECL positively influences AI-driven BMT (*H2*), reinforcing the argument made by Gafni and Levy^24^, who highlight the critical role of highly competent employees in ensuring the effective use of AI technologies in organizations. Employees with strong digital competencies and adaptability are better positioned to leverage AI tools, drive innovation, and facilitate business model transformation. Additionally, empirical evidence suggests that PED positively impacts AI-driven BMT (*H3*), corroborating the conclusions of Azhigali^2^ and Kumar et al.,^35^. This finding underscores that digitalization provides the foundational infrastructure for AI implementation, enabling organizations to maximize AI’s potential to reshape business models and achieve a sustained competitive advantage.

The results further demonstrate that TAS influences ECL (*H4*), consistent with the work of Savitha and Kumar^55^, who emphasize the crucial role of the speed of technological adoption in enhancing employee competency. Their research suggests that organizations that rapidly adopt new technologies enable employees to become familiar with innovations at an earlier stage, thereby accelerating skill development and career progression. Further, regarding H5, the analysis indicates that TAS also directly affects AI-driven BMT. This implies that, apart from increasing employee competency, the speed at which organizations embrace technology itself aids business model innovation by improving responsiveness and experimentation, as well as the early incorporation of AI into decision-making. This is consistent with Ji et al.,^30^ and Madanaguli et al.,^39^, who suggest that technology adoption is a primary driver of AI-driven BMT.

Finally, the results highlight the mediating role of ECL in the relationship between PED, TAS, and AI-driven BMT (*H6* and *H7*). This suggests that organizations that prioritize employee competency development are better positioned to leverage digitalization and technological adoption to facilitate AI-driven BMT. Consequently, investing in employee development should be a central component of effective digital transformation strategies.

### Theoretical implications

This study advances the RBV by demonstrating the critical mediating role of ECL in the relationships among PED, TAS, and AI-driven BMT. While RBV has traditionally focused on the strategic value of internal assets and capabilities in sustaining competitive advantage^5^, our findings suggest that ECL serves as a pivotal mechanism through which digitalization and technological adoption translate into meaningful business transformation. This outcome is consistent with recent studies on digital transformation, which suggest that technology assets often do not generate value for organizations without the human and organizational competencies required for their deployment^44^. This insight extends the conventional static perspective of RBV, which emphasizes resource possession, by highlighting the importance of continuous learning, skill development, and adaptive capability as organizational resources in their own right.

By integrating ECL into the RBV framework, this research improves understanding of how human capital interacts with digital transformation processes. In an age where AI transforms business models, success depends not only on having digital assets but also on how well the organization integrates them with the right people who can understand, use, and manipulate them to support the evolving business model. This idea is echoed by current studies on AI-driven business model innovations, which highlight the dynamic nature of AI transformations through management and organizational processes^31,38^.

This result implies that digital transformation goes beyond just a technological activity. It is an incredibly humanistic process that relies on employees’ learning and competency development as essential enablers of innovation and renewal. Consequently, the findings confirm the assumption that information systems cannot be understood only in terms of their technological nature. Instead, they should be examined as artifacts deeply embedded in a firm setting characterized by interdependence between technology and human agency. The use of a sociomaterial approach shows that information systems are not neutral instruments but are highly influenced by human cognition and interpretation, as well as by socio-materiality^59,66^.

The findings of this research contribute to the RBV by showing that resource development processes must be viewed in the context of exogenous technological factors^16^. Despite criticism of the RBV for its inward-looking approach to competitive advantage, the current findings suggest that it is necessary to consider how internal capability-building processes and technological dynamics relate to the maintenance of sustainable competitive advantage in the digital era^38^. In other words, the research findings are consistent with the theoretical framework of dynamic capabilities, which holds that companies must continually learn to recognize, exploit, and transform opportunities associated with AI use^25^. Such activities rely heavily on organizational employees’ ability to adapt and learn. Thus, failing to establish an AI competence development framework will prevent the company from maximizing the benefits of its technological investments and from making progress in evolving its business model^9^.

### Practical implications

This work offers valuable insights for both organizational leaders and policymakers, particularly within the Egyptian context, spanning six key sectors: manufacturing, services, technology, finance, retail, and healthcare. As AI-driven BMT becomes a strategic imperative, managers are advised to adopt a proactive approach to fostering TAS while simultaneously investing in ECL to maximize the benefits of digitalization.

A fundamental implication for managers is the need to embed a structured approach to technology adoption to ensure alignment with organizational objectives and employee readiness. Simply acquiring new technologies is insufficient; rather, firms must cultivate adaptive employees capable of effectively integrating AI-driven solutions into existing business processes. This requires not only the provision of technical training but also the development of a culture that encourages continuous learning, experimentation, and cross-functional collaboration. Organizations are advised to establish comprehensive upskilling programs, mentorship initiatives, and real-world AI application scenarios that enable employees to build hands-on expertise with emerging technologies. In this regard, adapting human resource management practices to the evolving social and technological landscape has been shown to enhance employee innovation and sustainability outcomes, further underscoring the strategic value of aligning HR systems with digital transformation objectives^48^.

Furthermore, decision-makers are advised to design a strategic roadmap for AI adoption that accounts for organizational infrastructure, digital maturity, and employee readiness. A structured implementation framework needs to be developed that integrates key considerations, including technology congruence with business strategy, anticipated skill gaps, and mechanisms for ongoing support and reskilling. Without such a framework, firms risk underutilizing technology, facing employee resistance, and experiencing misalignment between AI initiatives and long-term strategic goals.

Policymakers also have a role to play in fostering an enabling ecosystem for AI-driven transformation. Public-private collaborations, industry-academic partnerships, and sector-specific AI literacy programs can help bridge the skill gap and accelerate AI adoption across industries. By fostering initiatives such as tax incentives for workforce upskilling, grants for AI infrastructure development, and regulatory frameworks that encourage responsible AI integration, policymakers enable businesses to navigate the complexities of digital transformation.

Ultimately, this study underscores the need for a holistic approach to AI adoption—one that integrates technological investments with human capital development. Managers who recognize the interdependence between digital capabilities and workforce competency will be better positioned to drive sustained competitive advantage, ensuring that AI-driven BMT translates into meaningful innovation and long-term business growth.

### Limitations and future directions

This study relies on a quantitative, cross-sectional research design. While this approach allows for broad generalizability and statistical validation, it does not capture the dynamic, evolving nature of AI-driven BMT over time. Future research would benefit from adopting a mixed-methods approach or a purely qualitative methodology to gain deeper, context-specific insights into how organizations navigate AI adoption and competency development in practice.

The reliance on a non-probability, single-platform sampling approach via LinkedIn may introduce self-selection and coverage bias, potentially limiting the representativeness of the sample. Future studies are encouraged to adopt multi-source and probability-based sampling strategies to enhance the generalizability of the findings.

In addition, longitudinal studies are suggested, given that a more nuanced understanding of causal relationships can be gained by tracking how digitalization and AI adoption affect business model transformation over extended periods. Comparative analyses, such as pre- and post-implementation assessments of AI initiatives, could further clarify the mechanisms through which employee competency learning mediates the relationship between technological adoption and business transformation.

Another key consideration is the role of contextual factors that may influence the strength and direction of ECL’s mediating effect. Variables such as managerial support, organizational size, industry-specific structural conditions, and external market dynamics likely shape the ways in which PED and TAS interact with AI-driven BMT. Future research is suggested to explore the extent to which these factors moderate or mediate the observed relationships, thereby delineating the specific conditions under which employee skill development has the greatest impact on AI-driven transformation. Such investigations offer industry-specific insights, allowing for a more tailored approach to digital transformation strategies.

## Conclusions

This study sheds light on the critical role of ECL as a mediator in the relationship among PED, TAS, and AI-driven BMT. By integrating ECL into the RBV framework, the research highlights that AI adoption is not merely a technological shift but a deeply human-centric process in which employees’ skills and adaptability determine the success of business transformation efforts. Moreover, this study contributes to a deeper understanding of how AI adoption processes, if not adequately supported by human capabilities, themselves become sources of failure rather than success. In complex innovation environments, the lack of employee readiness leads to implementation pitfalls, misaligned expectations, and underperformance of AI initiatives. In contrast, organizations that view early challenges and setbacks as learning opportunities leverage employee competencies to reorient strategies and recover from innovation missteps. This highlights the dual role of AI as both an enabler of progress and a potential risk factor when organizational learning mechanisms are weak or underdeveloped. From this perspective, the capacity to learn from failure, especially failure associated with AI-enabled change, becomes a critical organizational competency.

The findings carry significant implications for both scholars and practitioners. For researchers, this study opens new avenues to explore the dynamic interplay between internal capabilities and external technological advancements, particularly in emerging economies where digital transformation is unfolding at varying speeds across industries. For managers and policymakers, the results underscore the importance of aligning AI initiatives with employee development strategies, ensuring that investments in technology are complemented by structured competency-building efforts. Without such alignment, organizations risk underutilizing AI’s potential and encountering resistance to change. The rapid evolution of AI and digitalization necessitates continuous inquiry into how organizations can sustain competitive advantage in this shifting landscape. What remains clear is that the AI-driven BMT is not simply a matter of acquiring cutting-edge technologies, but of cultivating a workforce capable of harnessing them effectively.

## Data Availability

The datasets generated and analysed during the current study are not publicly available due to participant confidentiality constraints, but are available from the corresponding author on reasonable request.
